# Circular RNA hsa_circ_000984 promotes colon cancer growth and metastasis by sponging miR-106b

**DOI:** 10.18632/oncotarget.21748

**Published:** 2017-10-10

**Authors:** Xiao-Wu Xu, Bo-An Zheng, Zhi-Ming Hu, Zhen-Yuan Qian, Chao-Jie Huang, Xi-Qiang Liu, Wei-Ding Wu

**Affiliations:** ^1^ Department of Gastrointestinal and Pancreatic Surgery, Zhejiang Provincial People's Hospital, Key Laboratory of Gastroenterology of Zhejiang Provincial People's Hospital of Hangzhou Medical College, Hangzhou, 310014, Zhejiang, China; ^2^ Department of Colorectal Surgery, Zhejiang Provincial People's Hospital, Hangzhou, 310014, Zhejiang, China; ^3^ Department of Hepatobiliary-Pancreatic Surgery, Zhejiang Provincial People's Hospital, Hangzhou, 310014, Zhejiang, China

**Keywords:** circular RNA, hsa_circ_000984, colon cancer, growth, miR-106b

## Abstract

Circular RNAs (circRNAs) as a novel type of noncoding RNAs (ncRNAs) are widely studied in the development of human various diseases, including cancer. Here, we found circular RNA hsa_circ_000984 encoded by the *CDK6* gene was remarkably upregulated in the tissues of colorectal cancer (CRC) patients and in the CRC cell lines. Moreover, high expression level of hsa_circ_000984 was significantly associated with advanced colorectal cancer. Further analysis revealed that hsa_circ_000984 knockdown could inhibit cell proliferation, migration, invasion *in vitro* and tumor formation *in vivo* in CRC cell lines. Mechanically, we found that hsa_circ_000984 may act as a competing endogenous RNA (ceRNA) by competitively binding miR-106b and effectively upregulate the expression of *CDK6*, thereby inducing a series of malignant phenotypes of tumor cells. Taken together, these observations suggest that the hsa_circ_000984 could mediate the expression of gene *CDK6* by acting as a ceRNA, which may contribute to a better understanding of between the regulatory miRNA network and CRC pathogenesis.

## INTRODUCTION

Colorectal cancer (CRC) is the common type of malignant tumor with the third occurrence and the fourth leading cause of related cancer death worldwide [1]. Nowadays, in spite of recent progresses in treatment for CRC including chemotherapy and radiation therapy, the prognosis of CRC remains relatively poor [2, 3]. In recent years, among the numerous molecules identified to involve in the development of CRC, noncoding RNAs (ncRNAs) have drawn increasingly focused for their aberrant expression features associated with tumor growth, or metastasis in CRC carcinogenesis [4–6].

In the past decades, whole genome and transcriptome sequencing technologies analysis revealed that a majority of mammalian genomes are transcribed to yield a large proportion of short or long RNAs transcripts with non-coding protein ability [7–10]. Among them, the circular RNAs (circRNAs) represents a re-discovered, abundant RNA molecular that are characterized by forming covalently closed-loop structures produced through an end-to-end formation during transcription [11–13]. Due to their abundance and stability, recent studies have found that circRNAs can be involved in a broad of biological activities by functioning as miRNA sponges and regulators of RNA binding protein in regulating protein-gene expression. The role of circRNAs as miRNA sponges to upregulate or downregulated gene expression in eukaryotes is the main mechanism in physiological and pathological processes [14]. Specifically, a firstly identified circRNA ciRS-7, also termed CDR1as, which harbors seventy miR-7-binding sites, was found to regulate miRNA in malignant tumor cells [15]. Similarly, another study has found evidence that the testis-specific circRNA (Sry) can serve as miRNA sponge for miR-138, influencing activity of miR-138 by binding to them [16, 17]. It is well established that certain miRNAs can exert important regulatory functions in cellular physiology by regulating gene expression, several lines of evidence implicate the remarkable functions of these circRNAs in multiple key biological processes [18, 19]. Moreover, increasing numbers of reports indicate that abnormal expressions of certain circRNAs are closely associated with a biological signature of various diseases including CRC [20–22].

Here, we focus on the circular RNA hsa_circ_000984 encoded by the *CDK6* gene. As a core member of the cyclin-dependent kinases (CDKs) family, CDK6 is necessary to drive progression of cell cycle passing from G1 to S phase by suppressing cell cycle inhibitors [23, 24]. Besides, deregulation of *CDK6* has also been reported to be associated with development and prognosis of various cancers [25–27]. In the present study, we set out to explore the potential role of circular RNAhsa_circ_000984 in the development and progression of CRC. We analyzed the hsa_circ_000984 expression level in CRC tissues, investigated the possible role of hsa_circ_000984 in CRC proliferation and cell cycle progression and explored the potential molecular mechanisms of hsa_circ_000984 in CRC cells.

## RESULTS

### Hsa_circ_000984 expression level in CRC tissues

**Figure 1:**
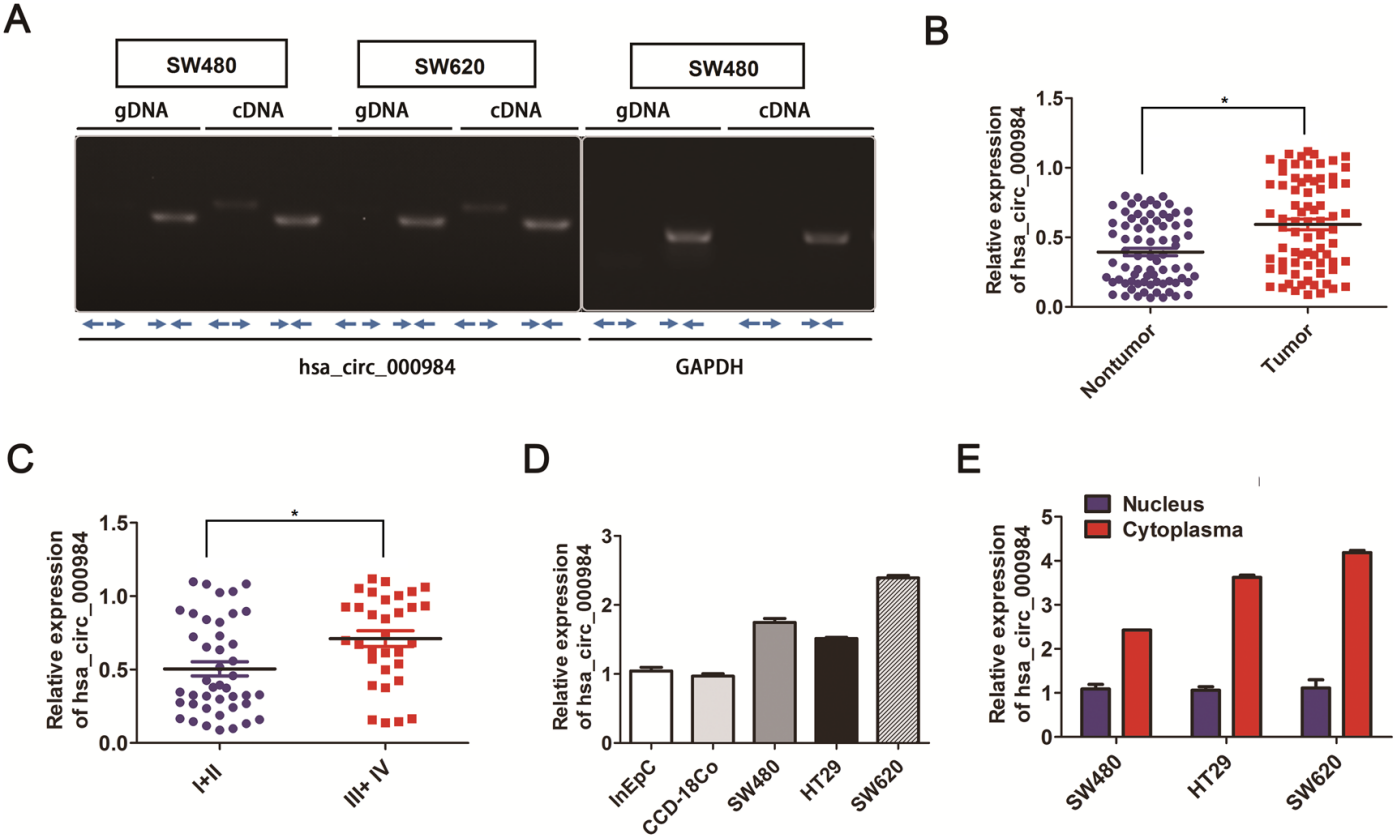
Analysis of hsa_circ_000984 expression in CRC **(A)** Hsa_circ_000984 could be significantly amplified in cDNA with divergent primers but did not amplify using genomic DNA as templates. Representative images of PCR products of hsa_circ_000984; GAPDH was used as a linear RNA control. **(B)** The results of hsa_circ_000984 expression level in CRC tissue samples and those matched colorectal nontumorous tissues were analyzed by qRT-PCR using divergent primer (**P<0.01, paired *t*-test). Data are shown as mean ± SD for three independent experiments (n=3). **(C)** The correlation analysis between hsa_circ_000984 expression level and TNM stage of CRC patients and hsa_circ_000984 expression was significantly associated with the advanced TNM stage (III+IV) (*P<0.05, logistic regression analysis). Data are shown as mean ± SD for three independent experiments (n=3). **(D)** qRT-PCR showed that hsa_circ_000984 was highly expressed in CRC cell lines of SW480, HT29 and SW620, compared with the normal colon-derived cell (CCD-18Co) and human intestinal epithelial cells (InEpC). **(E)** Levels of hsa_circ_000984 from nuclear and cytoplasmic fractions of CRC cell lines analyzed by qRT-PCR showed that hsa_circ_000984 was mainly enriched in the cytoplasmic fraction. Error bars indicate SD. *P<0.05, two-side Student’s *t*-test.

Based on circRNA databases [30, 31], the circRNA hsa_circ_000984 is generated from chromosomal region 7q (92462409-92463134), which contains one exon that create hsa_circ_000984 by head-to-tail splicing of exon [19]. We experimentally verified the hsa_circ_000984 exist in CRC cell lines using RT-PCR assays, with the divergent primers and opposite-directed primers. As shown in Figure 1A, the hsa_circ_000984 could be significantly detected using cDNA as templates with the above two set primers, confirming the circularization of hsa_circ_000984, while PCR assays using genomic DNA as templates with the divergent primers did not produce amplifications. *GAPDH* was used as a linear RNA control.

The hsa_circ_000984 expression patterns in 76 paired CRC tissue samples were further analyzed by qRT-PCR using divergent primer. Among them, we found the cirRNA to be significantly upregulated in CRC tissue samples than those matched colorectal nontumorous tissues (*P*<0.05) (Figure 1B). We further performed the correlation analysis between hsa_circ_000984 expression level and clinicopathological parameters (including age, sex, tumor size, TNM stage, tumor differentiation, and so) of CRC patients to evaluate the potential clinical diagnostic value of this circRNA (Supplementary Table 1). The result revealed that the expression of hsa_circ_000984 was significantly associated with the advanced TNM stage (III+IV) (*P*<0.05) (Figure 1C). To strengthen the significant quantification of hsa_circ_000984 in colorectal cancer, we enrolled another 32 colorectal cancer patient specimens to validate the expression of hsa_circ_000984 using qRT-PCR in paired colorectal cancer tissues and matched adjacent normal tissues. Consistent with the above results, expression level of hsa_circ_000984 was significantly upregulated in CRC tissue samples than those matched colorectal nontumorous tissues (*P*<0.05) and was correlated with the advanced TNM stage (III+IV) (*P*<0.05) (Supplementary Figure 1A and 1B).

### Hsa_circ_000984 expression in CRC cell lines

We further determined the expression levels of hsa_circ_000984in CRC cell lines. Compared with normal colon-derived cell (CCD-18Co) and human intestinal epithelial cells (InEpC), hsa_circ_0067934 was also highly expressed in the CRC cell lines (Figure 1D), implying the essential role in colorectal tumorigenesis. Additionally, cellular localization assay showed that hsa_circ_0067934 transcript was mainly enriched in cytoplasmic fraction, and cytoplasmic-located *GAPDH* and nuclear-localized *U6*: as expected, they were enriched in the cytoplasmic fraction and nuclear fraction, respectively (Figure 1E).

### Knockdown of hsa_circ_000984 significantly inhibited the proliferation of CRC cells

**Figure 2:**
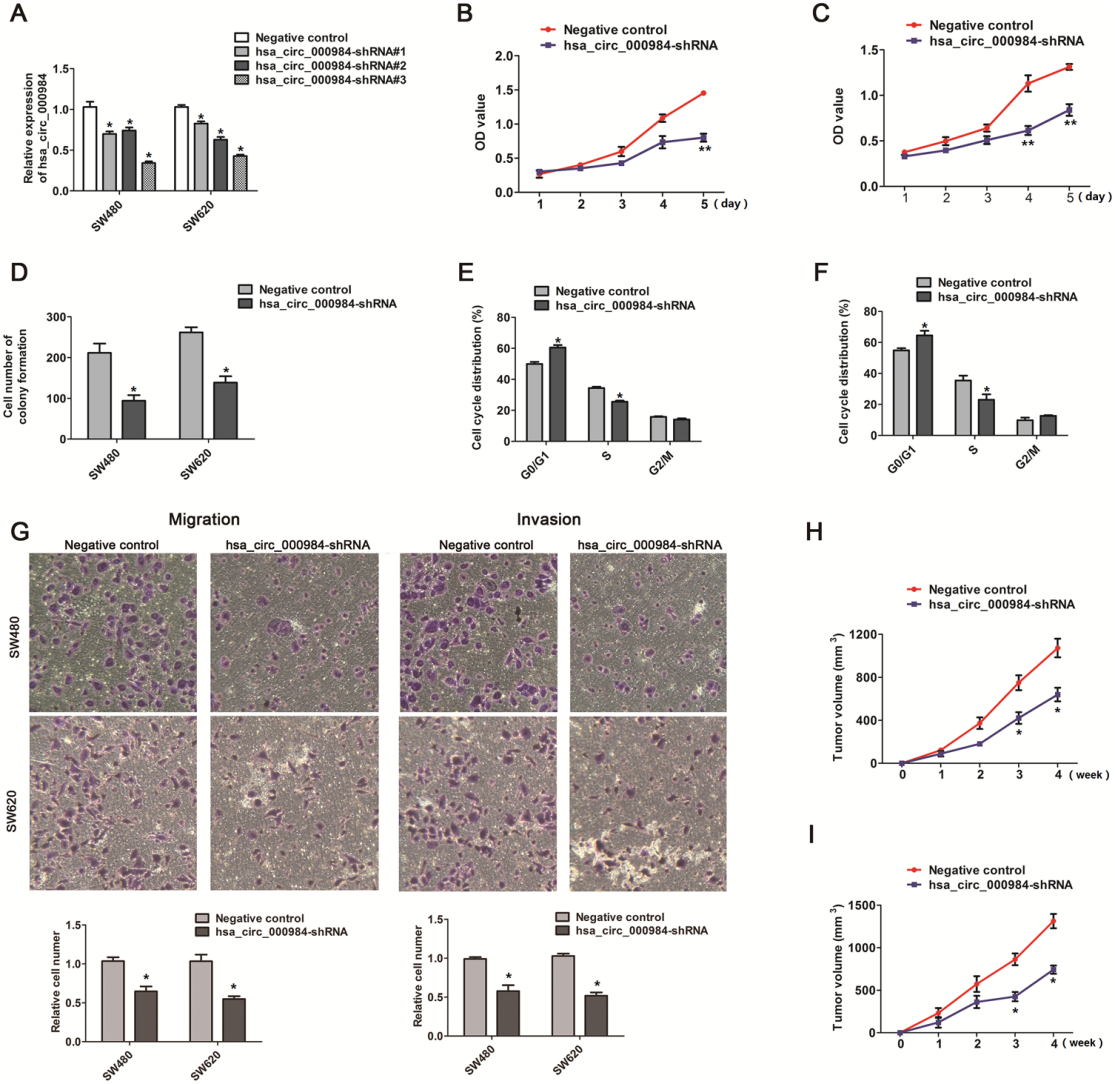
The effect of hsa_circ_000984 on cell proliferation, migration, invasion and tumor formation **(A)** qRT-PCR analysis of knockdown efficiency of hsa_circ_000984 in SW480 and SW620 cells transfected with shRNAs (shRNA#1, shRNA#2 and shRNA#3) targeting hsa_circ_000984 or a control shRNA (Negative control). Data are shown as mean ± SD for three independent experiments (n=3). *P<0.05, two-side Student’s *t*-test. **(B** and **C)** CCK-8 assay showed that CRC cells with stable expressing hsa_circ_000984 shRNA grew slower compared to the controls. **P<0.001, two-side Student’s *t*-test. Data are shown as mean ± SD for three independent experiments (n=3). **(D)** Colony formation assay showed that the knockdown of hsa_circ_000984 in CRC cells evidently reduced the numbers of colonies. **(E)** SW480 and **(F)** SW620 cells with stable expressing hsa_circ_000984 shRNA were mainly arrested in G0/G1 phase. **(G)** Migration and invasion assays for CRC cells with stable expressing hsa_circ_000984 shRNA and negative control. *P<0.05, two-side Student’s *t*-test. Data are shown as mean ± SD for three independent experiments (n=3). **(H)** SW480 and **(I)** SW620 cells with stable expressing hsa_circ_000984 shRNA were subcutaneously implanted in nude mice (n=8/group), and there was a statistical significance of tumor size between hsa_circ_000984 knockdown groups and control groups in the 28th day. *P<0.05, two-side Student’s *t*-test. All data indicate mean ±SD.

We performed CCK-8 assay and colony formation assay to analyze the effect of hsa_circ_000984expression on CRC cell growth. We knocked down hsa_circ_000984 expression in CRC cells using shRNAs, and the shRNA#3 was choose to use in the following investigation because the knockdown efficiency of hsa_circ_000984 by qPCR (Figure 2A). The knockdown of hsa_circ_000984 in CRC cells evidently reduced cell growth rate (Figure 2B and 2C). Colony formation assays was further confirmed that cells with stable expressing hsa_circ_000984 shRNA significantly reduced the numbers of colonies, compared with negative control (Figure 2D).

### Hsa_circ_000984 silencing blocks cell cycle progression in CRC cells

We speculated that the growth inhibition of CRC cells induced by hsa_circ_000984 silencing might be due to the disorder of cell cycle. To validate the possible roles of hsa_circ_000984 in cell cycle progression, we analyzed cell cycle phase distribution in CRC cells with stable expressing hsa_circ_000984 shRNA. In both tested cell types, we found that the cell cycle was mainly arrested in G0/G1 phase (Figure 2E and 2F) in the hsa_circ_000984-silienced CRC cell lines (60.5% *versus* 49.9%, *P*<0.05 for SW480 cells and 64.5% *versus* 54.8%, *P*<0.05 for SW620 cells), compared with the negative controls.

### Hsa_circ_000984 silencing suppress migration and invasion ability of CRC cells

We further performed migration and invasion assay to investigate whether the inhibition of hsa_circ_000984 suppresses the migration and invasion of CRC cells. Migration of cells was reduced >30% in CRC cells with stable expressing hsa_circ_000984 shRNA, compared with negative control cells (Figure 2G). Similarly, invasiveness of CRC cells with stable expressing hsa_circ_000984 shRNA was reduced >40%, suggesting that hsa_circ_000984 silencing represses CRC migration and invasion ability in CRC cells (Figure 2G).

### Hsa_circ_000984 silencing inhibits tumorigenesis in CRC xenograft model

Consistent with the above results, tumorigenesis assays showed that there was a statistical significance of tumor size in the 28th day between hsa_circ_000984 knockdown groups and control groups after two group cells were subcutaneously implanted in nude mice, and hsa_circ_000984 knockdown cells grew more slowly than negative control cells (639.667±63.63 mm3 versus 1072.67±86.48 mm3, *P*<0.01 for SW480 cells; and 742.81±48.40 mm3 versus 1314.067±84.67 mm3, *P*<0.01 for SW620 cells) (Figure 2H and 2I).

### Prediction of hsa_circ_000984 targeted circRNA-miRNA-mRNA network

**Figure 3:**
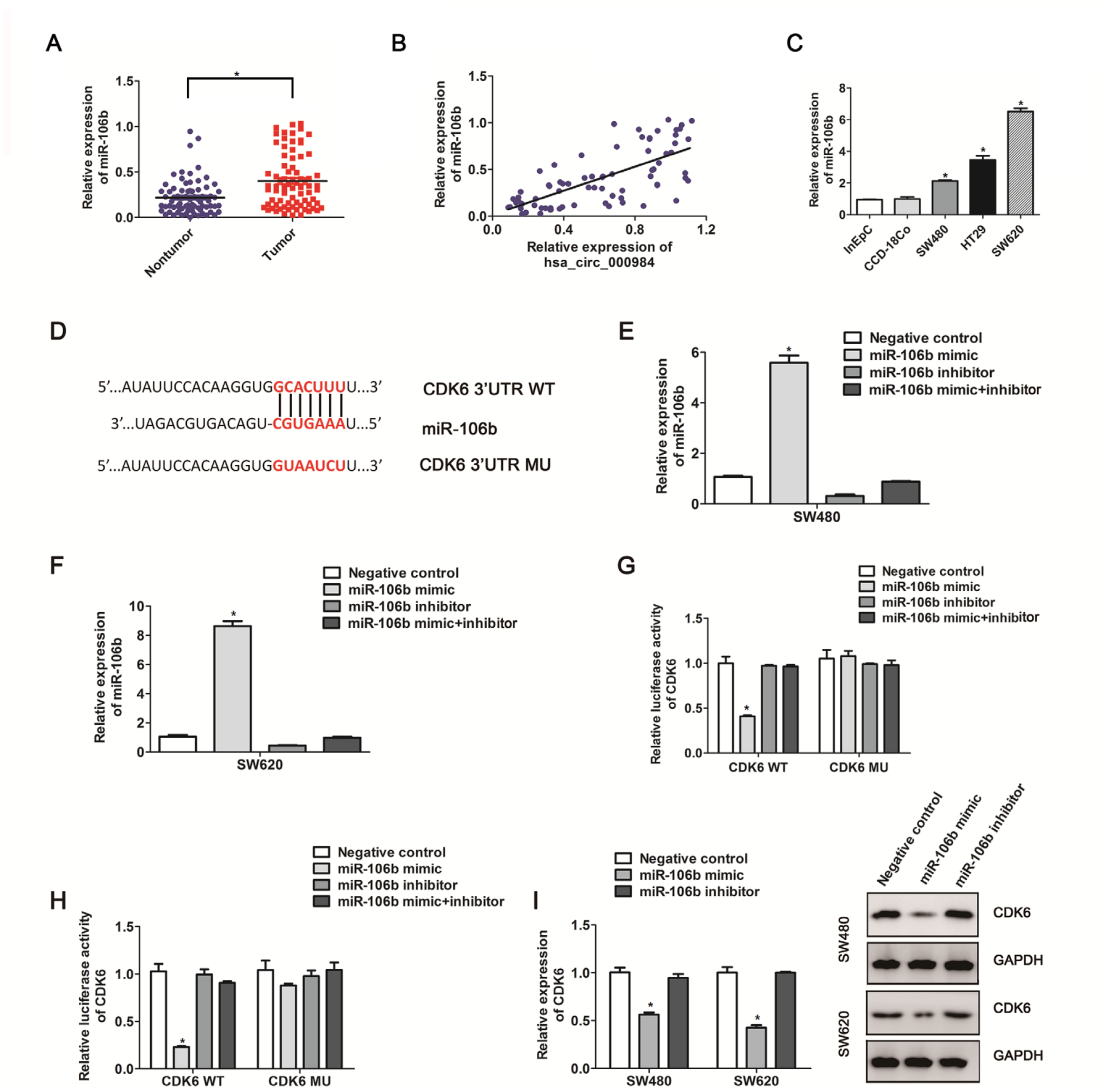
The correlation between CDK6 expression and miR-106b in CRC cells **(A)** MiR-106b expression in CRC tissue samples and those matched colorectal nontumorous tissues were detected by qRT-PCR. *P<0.05, paired *t*-test. **(B)** The correlation analysis between hsa_circ_000984 expression level and MiR-106b expression level in CRC samples (*P*<0.05, logistic regression analysis). **(C)** miR-106b expression was up-regulated in the CRC cell lines compared with CCD-18Co and InEpC cells. **(D)** Schematic representation of CDK6 3’-UTR and the miR-106b target site. The full-length of CDK6 3’-UTR containing miR-106b target site was inserted downstream of the firefly luciferase gene in psiCHECK2 to create the psiCHECK2-CDK 3’UTR-WT plasmid (Wt). The full-length of CDK6 3’-UTR deleted miR-106b-binding sequence was inserted downstream of the firefly luciferase gene in psiCHECK2 to create the psiCHECK2-CDK6 3’UTR-MU plasmid (MU). miR-106b expression levels in SW480 cells **(E)** and SW620 cells **(F)** co-transfected with miR-106b mimic or inhibitor. **(G** and **H)** luciferase activity was measured after CRC cell lines (SW480 and SW620) were transfected with CDK6 3’UTR-WT plasmid or CDK6 3’UTR-MU plasmid and the miR-106b mimic or inhibitor. **(I)** SW480 cells and SW620 cells were transfected with miR-106b mimic or inhibitor for 48 h. CDK6 mRNA level and protein level were measured by qRT-PCR and western blotting assays. Results are indicated as mean ±SD (n=3); *P<0.05, two-side Student’s *t*-test.

Until now, an amount of data have investigated that circRNA transcripts enriched with numerous miRNA-binding sites could function as miRNA sponges [17, 32, 33]. Next, we utilized the miRNA bioinformatics database TargetScan and miRanda, and identified 6 target miRNAs (miR-17, miR-20a, miR-93, miR-106a, miR-106b, miR-20b) for hsa_circ_000984. If hsa_circ_000984 indeed interacts with the 6 miRNAs, they both must be co-expressed. We then investigated the expression of miRNAs in CRC tissue samples and matched colorectal nontumorous tissues. We found that only miR-106b expression differed between the CRC tissues and those matched colorectal nontumorous tissues with statistical significance. As shown in Figure 3A and 3B, miR-106b was highly expressed in CRC tissue samples (*P*<0.01), and its expression was positively correlated with hsa_circ_000984 expression in CRC samples (R^2^=0.488, *P*<0.01), suggesting the co-expression of miR-106b and hsa_circ_000984 in CRC. Additionally, miR-106b levels were also higher in CRC cell lines than in control cells (Figure 3C).

Both circular hsa_circ_000984 and linear *CDK6* transcripts are encoded by the *CDK6* gene. To examine whether the miR-106b influences both mRNA and protein levels of *CDK6*, based on bioinformatics analysis, we found *CDK6* might be a target of miR-106b with the complementary binding sites with the *CDK6* 3’UTR (Figure 3D). MiR-106b level was determined by using qPCR after SW480 and SW620 cells were transfected with the miR-106b mimics and miR-106 inhibitors (Figure 3E and 3F). Next, we performed luciferase reporter assay to determine whether miR-106b directly target the *CDK6* 3’UTR by co-transfecting psiCHECK2-*CDK6*-3’UTR with miR-106b mimics. The luciferase activity was decreased observed about >72% in both SW480 cells and SW620 cells with the co-transfection of CDK6-3’UTR and miR-106b mimics, whereas mutant of nucleotides in the miR-106b-binding site of *CDK6* 3’UTR elicited no changes of the reporters to the introduction of miR-106b (Figure 3G and 3H). We further performed qPCR and immunoblot assays to determine the relationship between CDK6 and miR-106b levels. We found the mRNA and protein abundances of CDK6 consistently decreased with the introduction of miR-106b mimics into CRC cancer cells compared with negative controls (Figure 3I).

**Figure 4:**
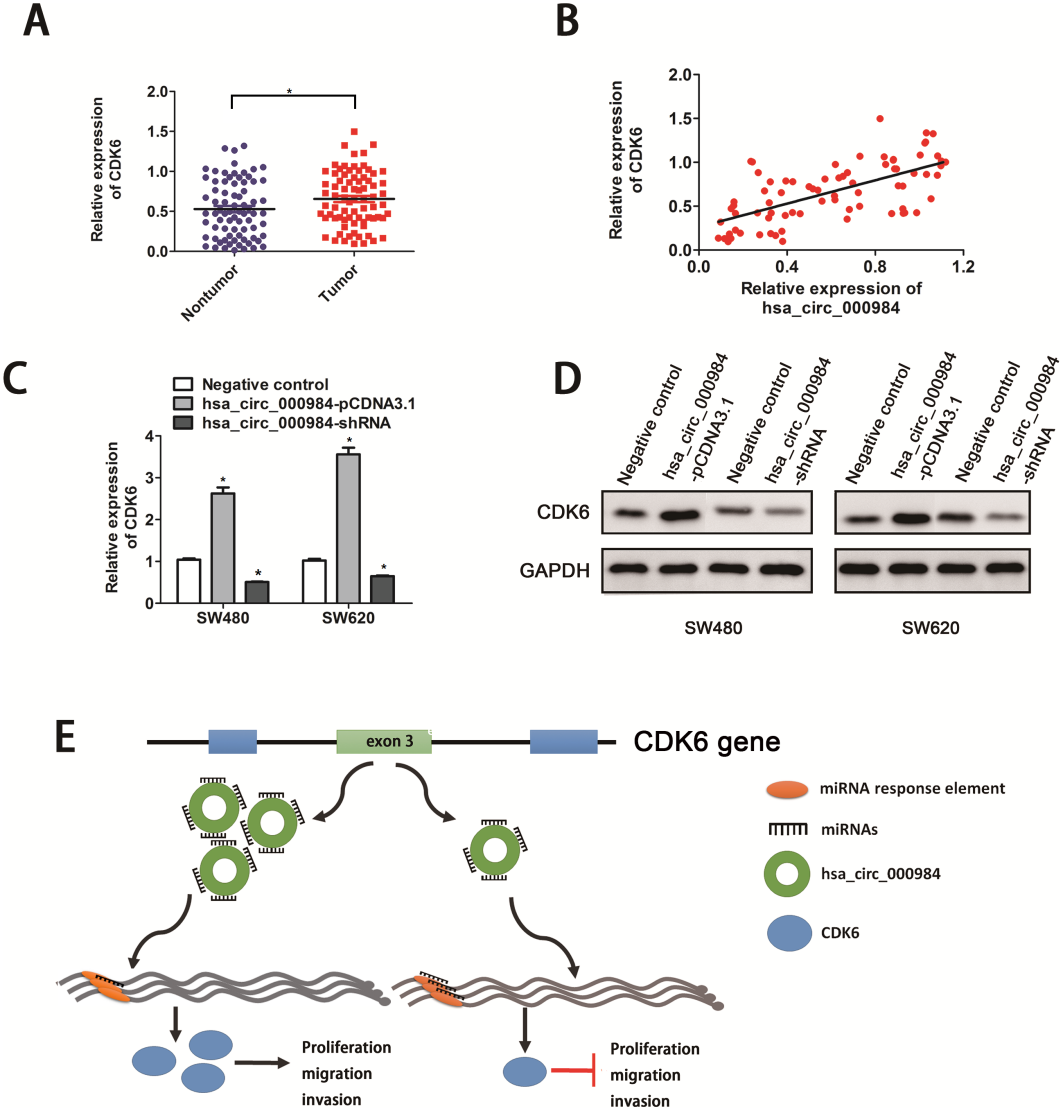
Hsa_circ_000984 may serve as miR-106b sponge to regulate its circRNA-miRNA-mRNA network **(A)** The expression levels of CDK6 in CRC tissue samples and their paired normal samples. The result showed that CDK6 expression in CRC tissue was significantly higher than those in corresponding nontumorous tissues (*P<0.05, paired *t*-test). **(B)** A significantly positive correlation was found of hsa_circ_000984 expression level with CDK6 expression level. CRC cell lines were transfected with hsa_circ_000984-shRNA or hsa_circ_000984-pCDNA-3.1 vectors, respectively. *P<0.05, two-side Student’s *t*-test. The expression of CDK6 were monitored by qRT-PCR **(C)** and western blotting assays **(D)**. **(E)** Schematic illustration of biological roles of hsa_circ_000984 in CRC carcinogenesis. Hsa_circ_000984 can bind to miR-106b as a miRNA sponge, exerting its function via regulating the downstream target CDK6, and knockdown of hsa_circ_000984 could inhibit CRC cell proliferation, migration, invasion *in vitro* and tumor formation *in vivo*, respectively.

We further examined whether misregulation of hsa_circ_000984 affected the level of the linear transcript of *CDK6* by RT-qPCR in CRC cancer. We found that *CDK6* expression level was dramatically upregulated in CRC tissue samples when compared to the adjacent normal tissues (*P*<0.05) (Figure 4A). Intriguingly, we explored the correlation of hsa_circ_000984 and *CDK6* levels in CRC tissues. A significant correlation was found of hsa_circ_000984 expression level with *CDK6* expression level (R^2^=0.413, *P*<0.01) (Figure 4B). Next, we assumed whether hsa_circ_000984 could act as miR-106b sponge to regulate its circRNA-miRNA-mRNA network. We knocked down and upregulated hsa_circ_000984 expression in CRC cells, respectively, and monitored expression of CDK6 by qPCR and western blotting assays. As illustrated in Figure 4C and 4D, decreased expression of hsa_circ_000984 in SW480 cells led to a markedly downregulation of CDK mRNA and protein levels. Conversely, overexpression of hsa_circ_000984 in CRC cells led to a significantly upregulation of CDK mRNA and protein levels, similar results was observed in SW620 cells.

## DISCUSSION

CircRNAs, largely disregarded as novel members of noncoding RNA family that are formed with out of order splice junctions precisely at their canonical linear counterparts [34]. Although these circRNAs have occasionally been reported for more than 20 years, they didn’t attract enough attention because of low abundance, and have been extensively considered as errors in transcripts splicing. In recent years, with the wide applications of high-throughput sequencing, a large number of circRNAs have been broadly identified in cells of various species [35–38]. Because of the unique formation of circRNAs by nonlinear reverse splicing, they are more stable and not easily degraded by RNase R. Thus, this remarkable feature provides them more advantageous as ideal diagnostic markers. Recently, many circRNAs have been shown to mainly function as miRNAs sponge by competing with the endogenous RNA network to directly affect the gene expression. Specifically, the most well-known circular RNA ciRS-7, which is spliced from the antisense transcript of the CDR1 gene, harbors extensive miR-7 binding sites to mediate miR-7 expression in tumor cells. Similarly, a study in esophageal squamous cell carcinoma, showed that cir-ITCH, has a sponging effect on miRNAs, stimulating ITCH levels, promoting tumor development and progression [21]. The cumulative evidence shows that circRNAs play an essential functional role in the carcinogenesis of cancer, including glioma [39], oral cancer [40], hepatocellular carcinoma [41] and colorectal cancer [42]. To our knowledge, this study is the first to investigate the biological roles of hsa_circ_000984 in CRC carcinogenesis.

Our study first demonstrated that hsa_circ_000984 was significantly up-regulated in CRC tissues and CRC cell lines, compared with adjacent normal tissues. Moreover, we found that high expression levels of hsa_circ_000984 in CRC patients were significantly related to advanced TNM stage (III+IV). We then explored the potential biological role of hsa_circ_000984 by shRNA mediated silencing. Through our series of experiments, we found that knockdown of hsa_circ_000984 inhibited the proliferation, migration, invasion of CRC cells *in vitro* and tumor formation *in vivo*, possibly by retarding cell cycle progression (G1 phase to S phase). In consistence with previous reports [19, 43], the hsa_circ_000984 is prominently located in cytoplasm. CircRNA are widely studied to play a strong regulatory function in carcinoma through a ceRNA mechanism by acting as a sponge of miRNAs [44, 45]. Hence, with the application of bioinformatics, we first found that hsa_circ_000984 may interact with miR-106b. Further experiments and analysis discovered that hsa_circ_000984 can bind to miR-106b as a miRNA sponge, contributing to the increase of downstream target CDK6 (Figure 4E). The well-known cyclin dependent kinases (CDKs) play essential role in the cell cycle regulation. Dysregulated activation of the CDK4/6 kinases is a hallmark of a variety of carcinomas [27, 46]. Alterations of CDK6 expression have been well documented in bladder cancer and colorectal cancer tissues, respectively [26, 47].

In summary, our data showed that expression levels of hsa_circ_000984 is upregulated in CRC tissues and cell lines. Besides, high hsa_circ_000984 expression is positively correlated with advanced CRC. Moreover, hsa_circ_000984 affected CRC cell growth, migration and invasion by competing with cell cycle-associated proteins for binding by miR-106b, indicating an essential role of hsa_circ_000984 in tumor and progression. The hsa_circ_000984 may be as a potential molecular markers for promising application perspectives of CRC.

## MATERIALS AND METHODS

### Tissue samples

76 paired CRC tumors and matched adjacent normal tissues were retrospectively selected from patients who undergoing surgical procedures at Zhejiang Provincial People's Hospital of Hangzhou Medical College. All patients agreed to sign informed consent in the study provided the detail clinic pathologic parameters including age, sex, histologic classification and tumor size. This study was approved by the Ethics Committee from the Zhejiang Provincial People's Hospital of Hangzhou Medical College.

### Cell culture

Human CRC cell lines (SW480, SW620 and HT29 cells) and normal colon-derived cell (CCD-18Co) and human intestinal epithelial cells (InEpC) included in this study were obtained from the Type Culture Collection of China Academic Science (Shanghai, China) and maintained at 37°C in 5% CO_2_ humidified atmosphere. These cells were cultured in RPMI Medium 1640 (Life Technologies, Carlsbad, CA, USA) supplemented with 10% fetal bovine serum (Corning, NY, USA) and antibiotics/antimycotics (Invitrogen, Carlsbad, CA).

### Immunoblot analysis

Protein analysis was performed as previously described [28]. Protein lysates extracted using RIPA cell lysis buffer (Beyotime Biotechnology) were electrophoresed on a 10% polyacrylamide gel (SDS-PAGE) and transferred to PVDF membranes (Hybond; Amersham Corp., Arlington Heights, IL, USA). Membranes were sequentially blocked with 5% milk for 1 hour and then probed with the indicated primary antibodies and the appropriate secondary antibodies (Cell Signaling Technology). Finally, blots were detected using a chemiluminescence reagent kit (Merck Millipore, Billerica, MA, USA).

### Quantitative RT–PCR (QRT-PCR)

The mRNA levels of hsa_circ_000984 and *CDK6* were detected by quantitative RT–PCR using a SYBR green PCR kit (Qiagen Korea, Seoul, Korea). Total RNA was extracted using the TRIzol reagent (CW Biotech, Beijing, China) according to the manufacturer's protocol. MiRNA expression was carried out using the TaqMan miRNA assay (Applied Biosystems, Foster City, CA). Relative quantification of gene expression was normalized by the 2^−ΔΔCt^ method relative to *GAPDH* and *RNU6B* which were used as qRT–PCR controls for genes and miRNAs. All experiments were performed in triplicate.

### Construction of stable cell lines

The anti-circ-RNA short hairpin RNAs (shRNAs) were synthesized and cloned into lentiviral vector (LV3) and were then packaged with Lentivector Packaging Plasmid mix (pGag/Pol, pRev, and pVSV-G) to establish stable cell lines as previously described [29]. Briefly, 293T Cells were cultured in a 6-well plate. The following day, cells were transfected with shRNAs plasmids and Lentivector Packaging Plasmids with Lipofectamine 2000 Reagent (Life Technologies). After 48h, the transfectants were selected with puromycin (Amresco, Cleveland, OH, USA) to gain the positive stably transfected clone with knockdown of hsa_circ_000984. The efficiency of hsa_circ_000984 knockdown was confirmed by qRT-qPCR.

### Plasmids and luciferase reporter assay

The full-length of *CDK6* 3’-UTR containing miR-106b target site and the full-length of *CDK6* 3’-UTR deleted miR-106b-binding sequence were inserted downstream of the firefly luciferase gene in psiCHECK2 to create the psiCHECK2-*CDK6* 3’UTR-WT plasmid (WT) and psiCHECK2-*CDK6* 3’UTR-MU plasmid (MU), respectively. The WT and MU plasmids subsequently were co-transfected into CRC cells with miRNAs mimics, or inhibitors along with control Renilla luciferase expression plasmid (phRL-TK) using Lipofectamine 2000. After 24h, luciferase and renilla signals were assayed using the Dual-luciferase reporter Assay System (Promega, Madison, WI, USA) according to the manufacturer's protocol.

### Cell proliferation assay, colony formation assay and cell cycle assay

A total of 2000 stably transfected cells in 100μl medium were seeded into each well of a 96-well plate. On days 0, 1, 2 and 3, 10μl of CCK-8 reagent (Dojindo Laboratories, Kumamoto, Japan) were added each well and proliferation was assayed by a microplate reader (Thermo Scientific Multiskan FC) to detected the absorbance value at 450 nm.

For colony formation assay, a total of 200 stably transfected cells were seeded into six-well plates and cultured for 2 weeks in standard conditions. Then the colonies were washed with PBS, fixed with methanol and then stained with crystal violet. Cell number was counted by a coulter counter.

For cell cycle assay 1×10^5^ stably transfected cells were washed in phosphate-buffered saline (PBS). Cellular DNA was stained with PI/RNase Staining Buffer (BD Biosciences, San Jose, CA, USA) and were then analyzed using flow cytometry (BD Biosciences).

### Tumor xenografts in nude mice

Negative control cells or treated cells with the indicated lentivirus vector with a concentration of 1×10^6^/ml diluted in 0.1 ml PBS were injected subcutaneously on the back flank of each mouse at day 0, respectively. Tumor size was measured with a caliper every 3 days. The tumor volume was calculated using the relationship (Volume=length×width^2^×0.5). Tumor growth rate was determined by averaging the volume of tumors of eight nude mice. All experiment procedures were carried out in accordance with the ethical standards under a protocol approved by the Committee on Animal Welfare of Zhejiang Provincial People's Hospital of Hangzhou Medical College.

### Bioinformatics analysis

TargetScan (http://www.targetscan.org/) and miRanda (http://www.microrna.org/) was used to predict the potential miRNAs binding sites in the hsa_circ_000984 and corresponding gene *CDK6* 3’UTR to study the possible crossing network among circRNA, miRNA and gene.

### Statistical analysis

All data are expressed as indicated by mean±SD. GraphPad Prism 5.0 (GraphPad Sotware, La Jolla, CA) was used to analyze the statistical data, where appropriate. The correlation of hsa_circ_000984 expression level and clinicopathological parameters were evaluated using t –test. The significance of hsa_circ_000984 expression level between CRC samples and matched colorectal nontumorous tissues were determined by the Student’ test, and statistical significance was set at *P*<0.05.

## SUPPLEMENTARY MATERIALS FIGURE AND TABLE


