# Supplementary material for: Circular RNA hsa_circ_000984 promotes colon cancer growth and metastasis by sponging miR-106b
Source: Oncotarget. 2017 Oct 10;8(53):91674–83. doi: 10.18632/oncotarget.21748 (PMC5710956; doi:10.18632/oncotarget.21748)
Supplement: Supplementary file 1 [file oncotarget-08-91674-s001.pdf]

Circular RNA hsa_circ_000984 promotes colon cancer growth and metastasis by sponging miR-106b

SUPPLEMENTARY MATERIALS

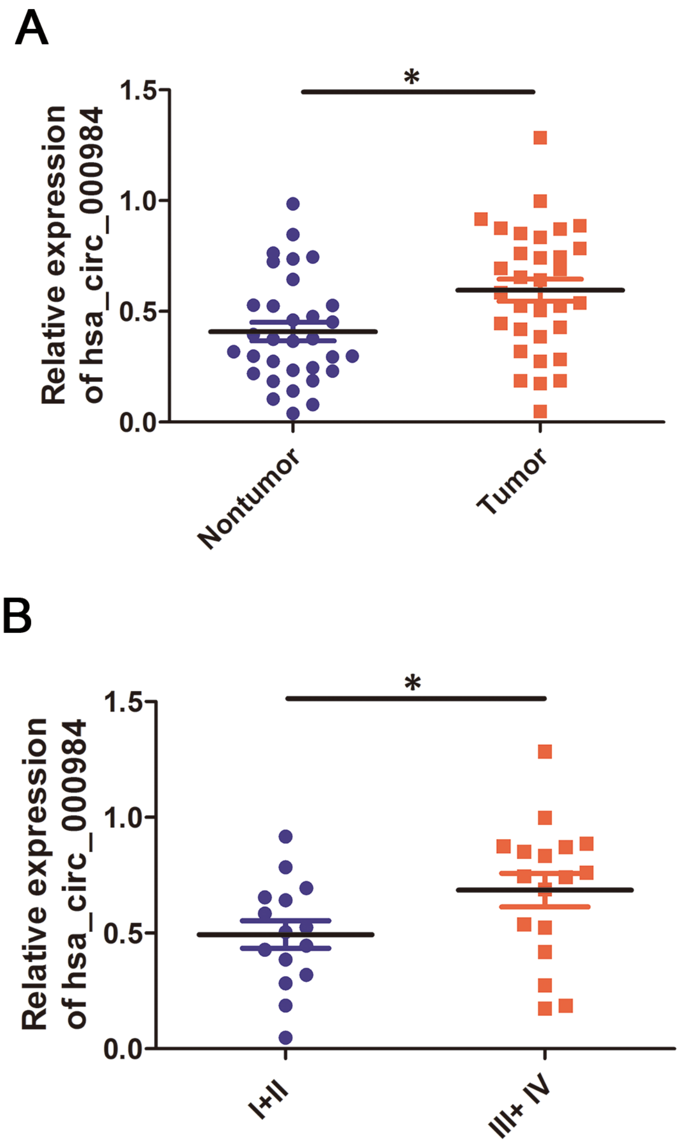

Supplementary Figure 1: **(A)** The expression level of hsa_circ_000984 in clinical colorectal cancerous tissues was significantly up-regulated compared with corresponding adjacentnormal tissues analyzed by qRT-PCR. **(B)** The expression level of hsa_circ_000984 in colorectal cancerous tissues was significantly higher in patients with a higher pathological stage (III+IV). *P<0.05.

Supplementary Table 1: Relationships between hsa_circ_000984 expression and the clinical clinicopathological characteristics of colorectal cancer patients.

Parameters	Total (76)	Expression of hsa_circ_000984				P_{value}*
		High		Low		
**Gender**						
Male	48	25	32.89	23	30.26	0.634
Female	28	13	17.11	15	19.74	
**Age**						
≤60	43	23	30.26	20	26.32	0.487
>60	33	15	19.74	18	23.68	
**Histological grade**						
Well/moderate	32	14	18.42	18	23.68	0.352
Other	44	24	31.58	20	26.32	
**Tumor size**						
≤2cm	39	21	27.63	25	32.89	0.336
>2cm	37	17	22.37	13	17.11	
**TNM stages**						
I+II	37	14	18.42	23	30.26	**0.035***
III+IV	39	24	31.58	15	19.74	

*Chi-square test.
*$P<0.05$
